# Urinary Markers of Glomerular Injury in Diabetic Nephropathy

**DOI:** 10.1155/2012/146987

**Published:** 2012-05-08

**Authors:** Abraham Cohen-Bucay, Gautham Viswanathan

**Affiliations:** ^1^Department of Medicine, St. Elizabeth's Medical Center, Tufts University School of Medicine, Boston, MA 02135, USA; ^2^Division of Nephrology, Tufts Medical Center, Boston, MA 02111, USA

## Abstract

Diabetic nephropathy, the leading cause of renal failure worldwide, affects approximately one-third of all people with diabetes. Microalbuminuria is considered the first sign and the best predictor of progression to renal failure and cardiovascular events. However, albuminuria has several limitations. Therefore, earlier, more sensitive and specific biomarkers with greater predictability are needed. The aim of this paper is to discuss the current literature on biomarkers of glomerular injury that have been implicated in diabetic kidney disease.

## 1. Introduction

Diabetes mellitus is a chronic disease that affects 366 million people worldwide (6.4% of the adult population) and is expected to rise to 552 million by 2030 [1]. People with diabetes require at least two to three times the health-care resources compared to people who do not have diabetes, and diabetes care may account for up to 15% of national health care budgets [2]. In 2008, 1.3 million deaths were associated with diabetes [3].

Diabetes results in both microvascular and macrovascular complications. Among the microvascular complications, diabetic kidney disease is one of the most serious, with significant impact on morbidity, mortality, and quality of life [4]. Diabetic nephropathy occurs in approximately one-third of all people with diabetes and is the leading cause of renal failure in developed and developing countries [3]. Death due to renal disease is 17 times more common in diabetics than in nondiabetics [5].

Clinically, the first sign of diabetic nephropathy is considered to be microalbuminuria. As the disease progresses, patients develop macroalbuminuria, and the kidney function declines until patients end up requiring renal replacement therapy [6].

Although microalbuminuria in diabetic patients is considered to be the best predictor of progression to end-stage renal disease [7] and cardiovascular events [8–10]; earlier, more sensitive and specific markers of kidney damage might help diagnose and treat diabetic nephropathy at an earlier stage to prevent the progression to renal failure.

Diabetic nephropathy affects all the kidney cellular elements, that is, glomerular endothelia, mesangial cells, podocytes, and tubular epithelia [11]. It is characterized by excessive accumulation of extracellular matrix (ECM) with thickening of glomerular and tubular basement membranes and increased amount of mesangial matrix, which ultimately progresses to glomerulosclerosis and tubulointerstitial fibrosis [11–13]. Multiple biomarkers in serum and urine have been studied that represent different mechanisms or structural damage, based on which they have been classified as markers of glomerular injury, tubular injury, oxidative stress, inflammation, and endothelial damage [5, 12].

Urinary markers of glomerular damage represent either, increased permeability to plasma proteins (albumin; transferrin), or increased excretion of extracellular matrix proteins (type IV collagen; fibronectin) [13]. The former is secondary to three main factors: loss of glomerular charge selectivity [14–20], loss of glomerular size selectivity [16, 18, 21–24], or increased intraglomerular pressure [25–27]. This paper will review the literature available regarding urinary biomarkers of glomerular injury associated with diabetic nephropathy.

## 2. Albumin 

Albumin, a 65-kDa protein produced in the liver, is the most abundant plasma protein in the body. The main functions of albumin are to regulate the oncotic pressure, to act as an acid/base buffer, and to mediate the transportation of metabolites, hormones, vitamins, and drugs [28]. 

In normal subjects, a small amount of albumin is filtered in the glomerulus, but almost all of it is reabsorbed by the tubules [29, 30]. Elevated urine albumin excretion (UAE) is considered a well-established marker of glomerular damage [12]. In addition, it is known that tubular dysfunction by itself may cause albuminuria owing to decreased reabsorption of filtered albumin [28]. 

The UAE is considered normal when it is less than 30 mg/day or 20 microg/min (normoalbuminuria). This threshold was determined because the UAE of 95% of “normal” patients falls below this value [8]. However, it has been recognized that the risk of cardiovascular events and renal morbidity is elevated also in subjects in the “high normal” range [8, 31–33]. 

Based upon the ability of dipstick to measure urine albumin, the UAE has been classified as microalbuminuria, when the UAE is between 30 and 300 mg/day or 20 and 200 microg/min; macroalbuminuria, when the UAE is above 300 mg/day or 200 microg/min. The rate of progression from micro to macroalbuminuria in type 2 diabetic patients is 2-3% annually [34].

Baseline albuminuria is the strongest predictor of end-stage renal disease (ESRD) for type 2 diabetic patients [7]. But, not all diabetic patients develop diabetic nephropathy. Approximately 20 to 40% of diabetic patients develop microalbuminuria within 10–15 years of diagnosis, whereas macroalbuminuria occurs within 15–20 years in 20–40% of patients [6]. The American Diabetes Association recommends screening with annual UAE on all type 1 diabetic patients with ≥5 years of disease duration and in all type 2 diabetic patients starting at diagnosis [35]. 

Microalbuminuria is not only a risk factor for chronic kidney disease (CKD) and ESRD, but it is also a strong predictor of total and cardiovascular mortality and cardiovascular morbidity in diabetic patients [8–10]. In patients with type 2 diabetes and nephropathy, albuminuria is the strongest risk marker for cardiovascular events [36]. 

Although albuminuria is widely used and is considered the best marker for renal damage in diabetic patients, certain limitations should be taken into consideration. First, not all patients with proteinuria will develop progressive renal dysfunction [6, 34]. Additionally, 30% of diabetic patients with renal impairment have normoalbuminuria [37]. Second, the cardiovascular and renal morbidity is elevated in the “high normal” range of UAE [8, 31–33]. Third, a number of variables affecting UAE lack standardization including urine collection methods, reporting of test results, reference intervals for albumin-to-creatinine ratio and lack of a complete reference system for urine albumin and creatinine measurements [38]. Finally, multiple markers of renal dysfunction, either tubular or glomerular, can appear before the detection of microalbuminuria, suggesting that microalbuminuria occurs once significant kidney damage has already occurred [12, 39]. 

## 3. Transferrin

Transferrin is a plasma protein very similar in weight (molecular weight 76.5 kDa) to albumin, but slightly larger (molecular radius 4.0 nm compared to 3.6 nm of albumin). It is less anionic than albumin with an isoelectric point (pI) one unit higher, therefore, expected to be filtered more readily through the glomerular barrier. Transferrin is the major iron-binding protein in the serum, and it transports ferric ions to all proliferative cells in the body [5, 12].

Among type 2 diabetic patients, urinary transferrin significantly increases with respect to the progress of biopsy proven glomerular diffuse lesions [40] and has been shown that some type 2 diabetic patients with diffuse glomerular lesions without microalbuminuria had microtransferrinuria [40]. Urinary transferrin excretion has also been correlated with the degree of interstitial fibrosis, tubular atrophy, and interstitial inflammatory cell infiltration [41]. 

Compared to healthy controls, transferrin excretion is higher in diabetic patients, even before they develop microalbuminuria [40, 42–48]. Because diabetic patients are more likely to have transferrinuria than albuminuria [44, 49–54], and because the albumin/transferrin ratio was significantly smaller in normoalbuminuric and microalbuminuric compared to macroalbuminuric patients, urinary transferrin is considered to be a more sensitive marker of glomerular damage in diabetic patients [44, 50–54]. Furthermore, increased urinary transferrin excretion predicts the development of microalbuminuria in type 2 diabetic patients with normoalbuminuria [55, 56]; in patients that already developed albuminuria, the urinary transferrin excretion has a linear relationship with UAE [39, 40, 42–44, 47, 50, 53, 57–60]. 

Urinary transferrin excretion is elevated in primary glomerulonephritis and other diseases that affect the glomerulus and is not specific to diabetic nephropathy [61, 62]. 

Although transferrinuria has been implicated as a cardiovascular risk factor, type 2 diabetic patients with both microalbuminuria and microtransferrinuria have a higher risk of ischemic heart disease than patients with microtransferrinuria only, suggesting that microalbuminuria may be a better predictor of ischemic heart disease than microtransferrinuria [60]. 

Urinary transferrin excretion is not correlated with glycemic control (hemoglobin A1c, fructosamine, and random glucose), supporting the hypothesis that transferrinuria is caused by intrinsic renal damage [42]. Nevertheless, glycemic control in newly diagnosed type 2 diabetic patients can effectively decrease transferrinuria [59]. Further evidence of transferrinuria as a marker of diabetic microvascular complications is the fact that urinary transferrin excretion is higher in type 2 diabetic patients with retinopathy [42, 44]. Conflicting results have been published regarding the correlation of urinary transferrin excretion and duration of diabetes [42, 44]. 

Similar to albumin [63], transferrin/creatinine ratio is associated with blood pressure control [42, 44]. However, only transferrinuria, and not albuminuria, has a correlation with diurnal changes in blood pressure [64]. 

In type 2 diabetic patients, transferrinuria precedes tubulointerstitial changes found on biopsy [41]. It is known that reabsorption of transferrin results in release of reactive iron [57], which can produce oxidative stress on the tubular epithelium. Several studies have shown that markers of proximal tubule damage (i.e., alpha-1-microglobulin and N-acetyl-beta-D-glucosaminidase (NAG)) and urinary transferrin excretion are associated in diabetic patients [40, 43, 44, 53, 59]. It is not clear if transferrinuria is secondary to decreased tubular reabsorption, or transferrin is the cause of tubular damage. 

According to two small nonrandomized trials, low-dose angiotensin receptor blockers (ARBs) seem to prevent the progression of transferrinuria, or even reverse it, independent of their antihypertensive effect. But further randomized controlled trials are needed to support that conclusion [48, 65]. 

## 4. Type IV Collagen

Type IV collagen is the main constituent of both glomerular and tubular basement membranes as well as the mesangial matrix [5, 66]. Elevated glucose levels stimulate type IV collagen synthesis and may reduce its breakdown by producing advanced glycosylation of proteins. As a consequence, increased deposition of type IV collagen has been noted in the glomerular mesangial matrix of diabetic kidneys with diffuse glomerulosclerosis [5, 67, 68]. Additionally, urinary type IV collagen excretion has been associated with mesangial expansion and tubulointerstitial and glomerular injury [69, 70]. The urinary excretion of type IV collagen correlates with the urinary excretion of other components of the glomerular basement membrane (GBM), including laminin [71]; markers of tubular damage, such as N-acetyl-beta-D-glucosaminidase (NAG) and alfa 1 microglobulin [66, 71, 72]. 

Higher urinary concentrations of type IV collagen have been found in diabetics compared to controls, even in normoalbuminuric subjects [66, 70, 71, 73–83], suggesting that type IV collagen could be an early predictor of diabetic nephropathy. In an Asian multicenter study of nearly 700 diabetic patients, Tomino et al. showed that the urinary excretion of type IV collagen in diabetic patients increased gradually as renal diseases progressed [82]. 

Multiple studies have shown that urinary excretion of type IV collagen in type 2 diabetics relates to UAE [66, 71, 72, 74, 78, 79, 82–84]. In contrast, patients with nondiabetic chronic glomerulonephritis do not show this relationship [66]. 

Although type IV collagen excretion is higher in nondiabetic chronic kidney disease compared to healthy controls, type 2 diabetic patients with evidence of kidney disease have a significantly higher type IV collagen/albumin ratio compared to patients with nondiabetic nephropathy [66, 71, 74], suggesting that urinary type IV collagen can help to differentiate diabetic versus nondiabetic nephropathy. 

In a prospective study, urinary type IV collagen was found to be more sensitive than albuminuria to detect renal damage in type 2 diabetic patients [72]. However, it has been reported that as many as 33% of microalbuminuric patients do not have increased urinary type IV collagen excretion [84]. In another study, Yagame et al. found that the area under the receiver operating characteristic (ROC) curve of albumin and type IV collagen was very similar, suggesting that UAE and urinary type IV collagen excretion have similar ability to detect early diabetic nephropathy [83].

In a follow-up study of 94 diabetic patients, Iijima et al. found that after 1 year, 25% of normoalbuminuric patients with increased urinary type IV collagen excretion developed microalbuminuria, and 75% stayed normoalbuminuric. The patients that stayed normoalbuminuric had a significant decrease in the urinary type IV collagen excretion, while the patients that developed microalbuminuria had a further increase in type IV collagen excretion [84]. 

Urinary type IV collagen excretion in type 2 diabetic patients is significantly associated with the duration of diabetes [71, 83]. Additionally, it is correlated with total serum cholesterol level [66] and inversely correlated with the reciprocal of serum creatinine [85]. However, it is not associated with diabetic retinopathy [71, 75]. Conflicting results have been published regarding the association of urinary type IV collagen excretion with blood pressure [71, 82, 84] and glycemic control [66, 71, 72, 74, 77, 79, 82–85]. 

Angiotensin-converting enzyme inhibitors (ACEIs) have been shown to decrease the type IV collagen urinary excretion in type 2 diabetic patients [86]. In contrast, low-dose ARBs failed to decrease the urinary type IV collagen excretion in a small nonrandomized, noncontrolled trial [87]. Further randomized, controlled studies are needed to conclude whether renin-angiotensin-aldosterone system blockade decreases the urinary excretion of type IV collagen. 

## 5. Fibronectin

Fibronectin, a high-molecular-weight protein, is an intrinsic component of the glomerular extracellular matrix. It is produced in the liver, vascular endothelia, and platelets. Fibronectin is involved in coagulation, platelet function, and tissue repair. In diabetes it may reduce erythrocyte deformity and filterability [5]. 

Urinary fibronectin excretion is higher in diabetic patients compared to controls, but the difference is only significant for macroalbuminuric patients [88, 89]. In diabetics, urinary fibronectin excretion is higher in patients with microalbuminuria compared to normoalbuminuria [88]. Additionally, urinary fibronectin levels correlate with the progression of biopsy proven glomerular diffuse lesions [90]. 

The excretion of urinary fibronectin degradation products correlates with UAE [91], and urinary fibronectin excretion has a weak negative correlation with creatinine clearance, mostly in patients with overt proteinuria [88, 90]. 

Urinary fibronectin excretion might be a useful biomarker of diabetic nephropathy, but further studies are needed to determine its relevance compared to albuminuria. 

## 6. Laminin

Laminin is a 900-kDa glycoprotein that is a normal component of basement membranes. It is considered that serum laminin cannot be filtered in the normal glomerulus, and the urinary laminin is derived from the kidneys [5]. It has been shown by immunohistochemistry that laminin is located in the mesangial expansion and thickened capillary basement membranes characteristic of diabetic nephropathy [92]. As expected, urinary laminin excretion correlates with the urinary excretion of type IV collagen, the main GBM constituent [71]. Because laminin is also found in the tubular basement membrane, it could be expected to find a relationship between urinary excretion of laminin and markers of tubular injury (i.e., NAG, alfa 1 microglobulin, beta 2 microglobulin, and kappa light chains), but conflicting results have been published regarding this correlation [71, 92, 93]. 

Urinary laminin excretion is higher in diabetic patients compared to healthy controls, even before the development of microalbuminuria [71, 75, 93]. However, there are conflicting results regarding the correlation of urinary laminin excretion with UAE [71, 92, 93]. 

Urinary laminin excretion increases with age, specifically in patients over 60 years of age [71, 92]. It is significantly correlated with the duration of diabetes [71], blood pressure [71], and glycemic control [71, 93]. 

Although urinary laminin excretion is higher in nondiabetic chronic nephropathy compared to controls, type 2 diabetic patients with evidence of nephropathy had significantly higher laminin/albumin ratio compared to patients with nondiabetic nephropathy [71], suggesting that urinary laminin excretion could help differentiate diabetic versus nondiabetic nephropathy.

Further studies are needed to determine the relevance of urinary laminin excretion in diabetic nephropathy. 

## 7. Glycosaminoglycans

Glycosaminoglycans (GAGs), with molecular weight ranging between 13 and 30 kDa, are important components of the extracellular matrix, cellular membranes, and endothelial glycocalyx. GAGs are involved in regulation of cell proliferation and differentiation, cell-to-matrix binding, cell-to-cell interaction, and regulation of interleukin-1 production. They are also a major component of basement membranes [94]; heparan sulfate, the most prevalent glycosaminoglycan in the GBM, has been recognized as the main anionic component of the GBM [95, 96]. In diabetes, there is a decrease in heparan sulfate content in the mesangial matrix and GBM, resulting in an alteration of the charge-selectivity of the glomerular capillaries, which may in part contribute to the proteinuria that characterizes diabetic nephropathy [5]. Hyperglycemia reduces the synthesis of GAGs by the glomerular endothelial cells decreasing the heparan sulfate content of the glycocalyx and thus increasing the passage of albumin through the glomerular capillary wall without affecting the interendothelial junctions [97]. Additionally, the systemic endothelial glycocalyx damage coincides with the development of microalbuminuria [98].

GAGs are also present in the tubular basement membrane and a correlation between urinary markers of tubular damage (beta-2 microglobulin, NAG, and Tamm-Horsfall protein) and urinary GAGs excretion has been shown [99–101]. Ueta et al. report an association between urinary GAGs excretion and the severity of the GBM lesion in diabetics with good glycemic control. While in poorly controlled patients it is associated with the severity of the tubulointerstitial lesion [102]. 

Multiple studies have described an increased urinary GAGs excretion compared to controls, even in normoalbuminuric patients [94–96, 99, 100, 102–111]. But one study found similar urinary GAGs excretions in normoalbuminuric diabetic patients and healthy controls [102]. 

Discordant results have been published regarding the correlation of urinary GAGs excretion and UAE. Most of the studies found that GAGs excretion increases as albumin excretion increases [96, 99, 100, 108, 109], two studies did not find a correlation [95, 106], and one study found a decrease in GAGs excretion as UAE increases [101]. Torffvit et al. [101] found a decrease in sulphated GAGs excretion (but not of GAGs/creatinine ratio) between normoalbuminuric and albuminuric type 1 diabetic patients. In this study they used methods to identify sulphated GAGs, while other studies have used methods that do not react with sulphated groups. Diabetes is known to induce the synthesis of hyaluronan, a nonsulphated GAGs [112]. Thus, increased urinary excretion of degraded hyaluronan can explain the increase levels of GAGs obtained in previous studies [109]. It is important to standardize the methods to measure and report urinary GAGs to conclude whether there is a correlation between urinary GAGs excretion and UAE. 

Diabetic patients with manifest nephropathy have increased urinary GAGs compared to patients with incipient nephropathy [94, 99, 108], and their sensitivity in patients with manifest nephropathy has been reported to be 100%, compared to 77% of albuminuria [99]. 

The prevalence of diabetic macroangiopathies in diabetic patients with elevated levels of urinary GAGs is significantly higher than in those with normal levels of urinary GAGs [100]. There is also a correlation between urinary GAGs excretion an diabetic neuropathy [99]. There are conflicting results regarding the correlation of urinary GAGs excretion and diabetic retinopathy; however, even the studies that reported a positive correlation show that the urinary GAGs excretion is not an independent risk factor for diabetic retinopathy [94, 99, 104, 110]. 

In diabetic patients, there is a correlation between urinary GAGs excretion and blood pressure [99, 101, 106, 108], but conflicting results have been published regarding their correlation with duration of diabetes [94, 96, 99, 106, 108, 110] and glycemic control [94, 96, 99, 101, 102, 106, 108, 109]. 

Urinary GAGs excretion could be a good marker for diabetic nephropathy and other complications of diabetes, but further studies and standardized methods of measurement of GAGs are needed before it is incorporated into clinical practice. 

## 8. Immunoglobulin G

Immunoglobulin G (IgG) is a protein synthesized and secreted by plasma cells than is mainly involved in the secondary immune response. It is larger than albumin, with a molecular weight of 150 kDa and molecular radii of 62 Å, compared to albumin 65 kDa and 36 Å, respectively [113]. 

Total urinary IgG excretion is higher in diabetic patients compared to controls, even before they develop microalbuminuria [45, 46, 48, 114–116]. Urinary IgG excretion in normoalbuminuric diabetic patients predicts the development of microalbuminuria [56] and, unlike UAE, it correlates with the progression of glomerular diffuse lesions [117]. Intense glycemic control [115] and low-dose losartan [48] have been shown to revert the increased IgG excretion in these patients. Diurnal changes in systolic blood pressure significantly correlates with urinary IgG excretion, but not with UAE [64]. 

Urinary IgG excretion correlates well with urinary excretion of orosomucoid (a marker of inflammation and endothelial damage), transferrin and ceruloplasmin [114], but it has a weak and nonlinear relationship with UAE, indicating that the urinary excretion of IgG rises later and moves slower than that of albumin [50, 114]. 

Apart form using the total IgG urinary excretion as a marker of glomerular damage, the relationship between the urinary excretion of IgG and its isoform IgG4 has been used more specifically, as a marker of glomerular charge selectivity impairment. In general, the more anionic a protein is, the more difficult to pass through the glomerular barrier. Because IgG and IgG4 have similar size (strokes radius of 55 Å), but IgG4 is more anionic (isoelectric point of IgG 7.3 and IgG4  5.8) [19], the difference in their urinary excretion would be explained only by a charge, and not size, selectivity defect. The selectivity index (SI) is the tool that has been more widely used to assess the ratio between IgG and IgG4. 

In microalbuminuric patients only IgG4 excretion is elevated, exemplified by a reduced SI in microalbuminuric compared to normoalbuminuric patients [16, 118]. While in macroalbuminuric patients excretion of both IgG and IgG4 are increased, shown by a similar IgG/IgG4 ratio but higher total IgG excretion in macroalbuminuric patients compared to microalbuminuric and normoalbuminuric patients [119, 120]. This suggests that the charge selectivity is lost in early diabetic nephropathy (microalbuminuric phase), which is difficult to evaluate in the macroalbuminuric phase because of the concomitant loss of size selectivity [14–16, 121–123]. 

The SI is not significantly different in normoalbuminuric diabetic patients compared to healthy controls [21, 39]; however, the urinary excretion of IgG4 and the SI has a significant correlation with UAE [17, 21, 39, 117, 124, 125]. Glycemic control [118], but not ACEI [125], increases the SI in type 1 diabetic patients with microalbuminuria. The clearance of IgG and IgG4 correlates with the duration of diabetes [15, 17]. 

The reduced SI in microalbuminuric patients does not correlate with markers of tubular injury (beta-2-microglobulin) [16], but a major disadvantage of measuring IgG and IgG4 is that both are reabsorbed in the tubules, causing the index to reflect the tubular and glomerular handling. Additionally, local production of IgG (e.g., prostate; seminal vesicles) and low-grade urinary tract infections can be other sources of error [21]. 

## 9. Ceruloplasmin

Ceruloplasmin, with a molecular weight of 151 kDa, is the major copper-carrying protein in the blood. It is more negatively charged than albumin [126] and therefore more difficult to be filtered by the glomerulus. 

Urinary ceruloplasmin excretion is higher in type 2 diabetic patients compared to controls [114], even in the normoalbuminuric phase [48, 115]. It correlates well with albumin excretion rate [126, 127] and predicts the development of microalbuminuria in normoalbuminuric patients [56]. Glycemic control [115] and low-dose losartan [48] revert the increased urinary ceruloplasmin excretion in normoalbuminuric patients. And diurnal changes in the systolic blood pressure significantly correlate with urinary ceruloplasmin excretion, but not with UAE [64]. 

The ceruloplasmin/creatinine ratio is higher in diabetic nephropathy compared to nondiabetic nephropathy patients [128]. It has been reported that urine ceruloplasmin/creatinine ratio has a sensitivity of 90-91%, specificity of 61–66% and 75% concordance, in diagnosing diabetic nephropathy [127, 128]. 

Ceruloplasmin is a promising marker of diabetic nephropathy, but further studies are necessary to characterize its value compared to UAE, especially in type 1 diabetics, since all the studies have been done in type 2 diabetics. 

## 10. Lipocalin-Type Prostaglandin D2 Synthase

Lipocalin-type prostaglandin D2 synthase (L-PGDS) is an enzyme-synthesizing prostaglandin D2 and a secretory protein of the lipocalin superfamily. It has similar chemical properties to albumin including anionic charge; however, it is much smaller in size (molecular weight 20–31 kDa), thus passing more easily through the glomerular capillary walls [129, 130]. L-PGDS is present in the peritubular interstitium and not in the tubular cells of nondiabetic patients while, in diabetic patients, it is present in the renal tubules [131]. 

Urinary L-PGDS excretion is higher in patients with any form of renal disease, except for males with IgA nephropathy, compared to controls. And has a sensitivity and specificity to diagnose renal disease of 67 and 86–93%, respectively [130]. It is more accurate than urinary type IV collagen, urinary markers of tubular injury (i.e., beta-2 microglobulin; NAG) and serum creatinine, but less accurate than UAE in diagnosing kidney disease [130]. Because decreased glomerular filtration rate decreases L-PGDS urinary excretion, it is thought to be useful in early stages rather than advanced kidney disease [130]. 

Urinary L-PGDS excretion is higher in type 2 diabetic patients compared to controls, even in those without albuminuria [131, 132], and independently correlates with the urinary protein excretion [132]. Urinary L-PGDS excretion is useful in predicting future development of albuminuria (>30 mg/gCr) in normoalbuminuric patients with a sensitivity and specificity of 56–59% and 75–88%, respectively [130]. Overall, L-PGDS is more accurate than urinary type IV collagen, beta 2 microglobulin, NAG, and serum creatinine in predicting proteinuria [130]. Thus, urinary L-PGDS excretion is useful to detect early renal damage in normoalbuminuric patients. Combined with albumin, it increases the power to detect diabetic nephropathy in those patients already proteinuric [130]. Glycemic control decreases L-PGDS excretion to the normal range in normoalbuminuric patients [131]. 

In type 2 diabetic patients, the presence of higher L-PGDS excretion is independently associated with history of cardiovascular disease [133]. 

Urinary L-PGDS has been mainly studied in type 2 diabetic patients. It is a promising novel urinary marker of kidney disease, but further studies are needed to define its role in diagnosing diabetic nephropathy. 

## 11. Immunoglobulin M

Immunoglobulin M (IgM), secreted by plasma cells, is the largest antibody in the human circulatory system. Due to its large molecular radius (120 Å), the appearance of IgM in the urine indicates an increased density of large, highly nonselective pores (“shunts”) in the glomerular capillary wall, which implicates a severe size-selectivity defect [18, 116, 134, 135]. 

Increased urinary IgM excretion in patients with nondiabetic glomerular disease is associated with high degree of fibrosis and global glomerulosclerosis [134]. Furthermore, high urinary IgM excretion is a better predictor of decline in kidney function than albuminuria in these patients [134, 136, 137]. 

Increased urinary IgM excretion and IgG2/IgG4 ratio in macroalbuminuric type 2 diabetic patients compared to type 1 diabetic patients suggests that the proteinuria in type 2 diabetes is due to size-selectivity defects, while charge selectivity defects account for the proteinuria in type 1 diabetes [116]. 

Urinary IgM excretion is higher in macroalbuminuric type 2 diabetic patients compared to healthy controls but does not correlate with UAE or urinary alfa-1-microglobulin excretion (a marker of tubular injury) [116]. Increased urinary IgM excretion, independent of UAE, predicts cardiovascular mortality and progression to ESRD in diabetic patients [135, 138]. 

Urinary IgM excretion has not been studied as an early marker of diabetic nephropathy since it is associated with severe injury of the glomerular capillary wall. However, it is a promising marker that may predict the eventual need for renal replacement therapy and cardiovascular mortality. Urinary IgM has been mostly studied in small trials in the Nordic population and large trials in other ethnic groups are needed before it is implemented in clinical practice. 

## 12. Conclusion

The current gold standard for detection and prediction of diabetic nephropathy and cardiovascular risk is albuminuria; however, it has several limitations. Makers that offer higher sensitivity and specificity for earlier detection of diabetic kidney disease and more accurate prediction of the progression to ESRD are needed. We reviewed nine biomarkers of glomerular injury implicated in diabetic kidney disease (Table 1). 

Considering the results of the studies evaluating the biomarkers reviewed here, it is appealing to start utilizing them in clinical practice. However, the majority of publications reviewed are small cross-sectional studies, and there are only a handful of longitudinal studies. Moreover, biomarkers only have clinical value if the results are reproducible, and none of the biomarkers reviewed here have been studied in more than 2 longitudinal trials. Hence, their clinical applicability needs to be confirmed in high-quality validation studies. 

Furthermore, the majority of these studies, when reporting prediction of outcomes, use odds ratios or hazard ratios, which are inaccurate to predict the risk for individual subjects. Hellemons et al. in a systematic review of longitudinal trials, have suggested the use of area under the ROC curve, positive/true-positive fractions, net reclassification improvement, integrated discrimination improvement, or the discriminative likelihood ratio as better methods to validate new markers [4]. 

Another methodological issue is the use of “transition in albuminuria class” as an endpoint. For example, a patient who had an increase in UAE from 25 to 35 mg/day would be recognized as transitioning from normoalbuminuria to microalbumiruria, while a patient with increase from 35 to 295 mg/day, would not be considered as a progressor. We have to remind ourselves that the classification of albuminuria was based on the ability of dipstick to measure urinary albumin and not the association with disease. Furthermore, it is known that risk of cardiovascular events and renal morbidity is elevated in subjects in the “high normal” range of proteinuria [8, 31–33]. A more accurate way to report changes in albuminuria would be to assess the absolute changes in UAE. 

Although many of the biomarkers reviewed here are promising, current data prevents us from making clear recommendations regarding their possible clinical use. Efforts on biomarker research should be directed at both new biomarker discovery and validation of published biomarkers on good quality, long-term, large longitudinal trials. Eventually, efforts should be made to develop a biomarker panel that is able to reliably assess diabetic nephropathy.

## Figures and Tables

**Table 1:** Overview of biomarkers of glomerular injury in diabetic nephropathy*.

Marker	DM1	DM2	Prior to MA**	Predicts MA***	UAE	Diabetic retinopathy	CV risk
Transferrin	+	+	+	+	+	+	+^*τ*^
IV-C	+	+	+		+	−	
Fibronectin	+	+			+		
Laminin	+	+	+		+/−		
GAGs	+	+	+		+/−	−	+
IgG	+	+	+	+	+		
Ceruloplasmin	−	+	+	+	+		
L-PGDS	−	+	+	+	+		+
IgM	+	+					+

DM1: studies in type 1 diabetic patients, DM2: studies in type 2 diabetic patients, MA: microalbuminuria, UAE: correlation with urine albumin excretion, CV: cardiovascular, IV-C: type IV collagen, GAGs: glycosaminoglycans, IgG: immunoglobulin G, L-PGDS: Lipocalin-type prostaglandin D2 synthase, IgM: immunoglobulin M. *: an empty space means not enough data published, **: presence prior to microalbuminuria, ***: predicts the development of microalbuminuria, “+/−”: conflicting results are published,  *τ*: transferrin is correlated with cardiovascular risk, but albumin is a better predictor of ischemic heart disease.
